# Hierarchical Surface Pattern on Ni‐Free Ti‐Based Bulk Metallic Glass to Control Cell Interactions

**DOI:** 10.1002/smll.202310364

**Published:** 2023-12-18

**Authors:** Fei‐Fan Cai, Andreu Blanquer, Miguel B. Costa, Lukas Schweiger, Baran Sarac, A. Lindsay Greer, Jan Schroers, Christian Teichert, Carme Nogués, Florian Spieckermann, Jürgen Eckert

**Affiliations:** ^1^ Department of Materials Science Chair of Materials Physics Montanuniversität Leoben Jahnstraße 12 Leoben A‐8700 Austria; ^2^ Erich Schmid Institute of Materials Science Austrian Academy of Sciences Jahnstraße 12 Leoben A‐8700 Austria; ^3^ Departament de Biologia Cel·lular Fisiologia i Immunologia Universitat Autònoma de Barcelona Cerdanyola del Vallès Bellaterra 08193 Spain; ^4^ Department of Materials Science & Metallurgy University of Cambridge Cambridge CB3 0FS UK; ^5^ Department of Mechanical Engineering and Materials Science Yale University New Haven CT 06511 USA; ^6^ Department Physics Mechanics and Electrical Engineering Chair of Physics Montanuniversität Leoben Franz‐Josef‐Strasse 18 Leoben A‐8700 Austria

**Keywords:** biocompatibility, biomaterials, bulk metallic glass, patterning, thermoplastic forming, titanium alloys, topography

## Abstract

Ni‐free Ti‐based bulk metallic glasses (BMGs) are exciting materials for biomedical applications because of their outstanding biocompatibility and advantageous mechanical properties. The glassy nature of BMGs allows them to be shaped and patterned via thermoplastic forming (TPF). This work demonstrates the versatility of the TPF technique to create micro‐ and nano‐patterns and hierarchical structures on Ti_40_Zr_10_Cu_34_Pd_14_Sn_2_ BMG. Particularly, a hierarchical structure fabricated by a two‐step TPF process integrates 400 nm hexagonal close‐packed protrusions on 2.5 µm square protuberances while preserving the advantageous mechanical properties from the as‐cast material state. The correlations between thermal history, structure, and mechanical properties are explored. Regarding biocompatibility, Ti_40_Zr_10_Cu_34_Pd_14_Sn_2_ BMGs with four surface topographies (flat, micro‐patterned, nano‐patterned, and hierarchical‐structured surfaces) are investigated using Saos‐2 cell lines. Alamar Blue assay and live/dead analysis show that all tested surfaces have good cell proliferation and viability. Patterned surfaces are observed to promote the formation of longer filopodia on the edge of the cytoskeleton, leading to star‐shaped and dendritic cell morphologies compared with the flat surface. In addition to potential implant applications, TPF‐patterned Ti‐BMGs enable a high level of order and design flexibility on the surface topography, expanding the available toolbox for studying cell behavior on rigid and ordered surfaces.

## Graphical abstract

A hierarchical structure integrating nano‐scale protrusions on micro‐scale protuberances is achieved on Ni‐free Ti‐based bulk metallic glasses via a two‐step thermoplastic forming technique. The patterned materials preserve advantageous mechanical properties and biocompatibility from the as‐cast materials. However, the surface features change the cell morphology. Besides implant applications, this work realizes a strategy for studying cell behavior on rigid ordered surfaces.

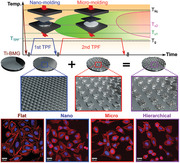

## 1 Introduction

Titanium‐based bulk metallic glasses (Ti‐BMGs) have recently attracted substantial interest in biomedical applications. The high titanium content of these alloys gives them intrinsic biocompatibility.^[^ ^1^, ^2^, ^3^ ^]^ In addition, owing to their amorphous nature, Ti‐BMGs have attractive properties such as high corrosion resistance, wear resistance, lower Young's modulus, higher elasticity, and higher specific strength than traditional crystalline Ti‐alloys, making them potential biomaterials for dental and orthopedic applications.^[^ ^2^, ^3^, ^4^, ^5^, ^6^, ^7^ ^]^ The reduced Young's modulus and enhanced elastic behavior are expected to alleviate stress‐shielding effects when contacting bone tissues.^[^ ^8^, ^9^ ^]^ Indeed, many efforts have been devoted to the development of β‐type titanium alloys achieving low Young's modulus below 80 GPa for crystalline materials.^[^ ^10^, ^11^, ^12^, ^13^ ^]^ In amorphous materials, many Ti‐BMGs, such as Ti─Ni─Cu and Ti─Zr─Cu─Ni alloy systems, rely on nickel to improve their glass‐forming ability (GFA).^[^ ^6^, ^14^ ^]^ Several studies have shownthat nickel ions can induce harmful effects on the human body, such as cutaneous inflammations and carcinogenicity.^[^ ^15^, ^16^ ^]^ Ion release is a by‐product of corrosion, which in turn is generally facilitated by contact with body fluid.^[^ ^6^, ^15^ ^]^ Without Ni, Ti─Zr─Cu─Pd BMG systems have improved biocompatibility and sufficient GFA for real‐life applications.^[^ ^14^, ^17^, ^18^ ^]^

BMGs can be processed by the thermoplastic forming (TPF) technique, another advantage for amorphous structures.^[^ ^19^, ^20^ ^]^ When BMGs are heated to their supercooled liquid region , the region between glass transition temperature (*T*_g_) and crystallization temperature (*T*_x_), the material can be deformed by viscous flow.^[^ ^17^, ^19^, ^20^, ^21^ ^]^ Viscous flow deformation can be used to shape the entire feedstock and/or fabricate surface features on BMGs.^[^ ^19^, ^20^, ^22^, ^23^, ^24^ ^]^ Thus, TPF techniques can be used to, for example, manufacture screws from BMG rods. As an improved variant of Ti─Zr─Cu─Pd BMGs, Ti_40_Zr_10_Cu_34_Pd_14_Sn_2_ (at%) BMG has more potential to be processed with TPF compared to the base composition Ti_40_Zr_10_Cu_36_Pd_14_ BMG.^[^ ^17^, ^20^ ^]^ Replacing 2 at% of Cu with Sn in Ti_40_Zr_10_Cu_36_Pd_14_ BMG effectively slows the kinetics of crystallization in the Ti_40_Zr_10_Cu_34_Pd_14_Sn_2_ BMG, resulting in an enhanced GFA and a wider supercooled liquid region.^[^ ^17^, ^25^, ^26^ ^]^ Ti_40_Zr_10_Cu_34_Pd_14_Sn_2_ BMG has a critical diameter of 12 mm, which is sufficient for most dental and orthodontic applications, such as fixtures, screws, abutments, brackets, hooks, and ligatures.^[^ ^2^, ^14^, ^26^ ^]^

In terms of biomedical science, the surface morphology of the implants affects osseointegration and anti‐biofilm properties.^[^ ^9^, ^27^, ^28^, ^29^ ^]^ Several studies prove that cells can sense their environment and convert mechanical stimuli such as stiffness and topography into biochemical signals to regulate cell response and function; this process is known as mechanotransduction.^[^ ^29^, ^30^, ^31^, ^32^, ^33^, ^34^, ^35^ ^]^ Hence, the surface topography of the implant can induce changes in cell morphology and further impact cell fate.^[^ ^1^, ^6^, ^9^, ^27^, ^35^, ^36^, ^37^ ^]^ It was demonstrated how the density of nano‐protrusion controls responses of human mesenchymal stem cells, in which the dense pattern (1.2 µm spacing) inhibited cell spreading and directed cells to become adipocyte‐like while the sparse pattern (5.6 µm spacing) induced cells to become osteoblast‐like cells.^[^ ^38^, ^39^ ^]^ Moreover, different cell types might respond differently to the same topography. Padmanabhan et al. showed that fibroblasts could detect as small as 55 nm nano‐patterns.^[^ ^40^ ^]^ In comparison, macrophages failed to detect nano‐patterns of smaller 150 nm and responded to a feature size of 200 nm.^[^ ^40^ ^]^ Finally, engineering the surface topography can improve implant grip and avoid loosening to save patients from pain.^[^ ^41^, ^42^ ^]^

Surface patterned Ni‐free Ti‐BMGs bridge the gap between materials science and biomedical engineering, but literature reports are limited. Regarding hierarchical structures on metallic glasses, Hasan et al. implemented hierarchical patterning on Pt_57.5_Cu_14.7_Ni_5.3_P_22.5_ BMG.^[^ ^43^ ^]^ For Ti‐BMGs, Gong et al. have decorated surfaces with 400 nm‐diameter nanorods on Ti_45_Zr_20_Be_30_Fe_5_ BMG.^[^ ^44^ ^]^ However, beryllium which is part of the latter alloy has high cytotoxicity and should be avoided in implants.^[^ ^45^, ^46^ ^]^ Sarac et al. generated hierarchical square bumps with a maximum height of 5 µm, where the micro‐forming kinetics were compared with Zr_48_Cu_36_Al_8_Ag_8_.^[^ ^47^, ^48^ ^]^ For the above‐mentioned Ti_40_Zr_10_Cu_34_Pd_14_Sn_2_ BMG, Bera et al. demonstrated a circular bump pattern with roughly 500 µm diameter and 24 µm height.^[^ ^17^ ^]^ Cai et al. managed to micro‐patterned square protuberances with 5 µm in length and around 1.6 µm in height.^[^ ^20^ ^]^ However, hierarchical structures integrating micro‐ and nano‐patterns at the same surface have not been achieved on Ti‐based BMGs yet.

Here, for the first time, nano‐patterns and hierarchical structures are created on the surface of Ni‐free Ti‐based BMGs. On Ti_40_Zr_10_Cu_34_Pd_14_Sn_2_ BMG, we demonstrate the nano‐pattern with a dragonfly‐eye‐like microstructure featuring 400 nm protrusions and a raspberry‐shaped hierarchical structure by superimposing 400 nm diameter nano‐protrusions on 2.5 µm square protuberances. The influences of various TPF conditions on structures and mechanical properties are studied. Biocompatibility of Ti_40_Zr_10_Cu_34_Pd_14_Sn_2_ BMG is investigated using Saos‐2 cell lines on four different surface topographies: flat, micro‐patterned, nano‐patterned, and hierarchical‐structured surfaces. The influence of surface patterns on cell morphology is inspected by scanning electron microscopy (SEM) and confocal laser scanning microscopy (CLSM) and determined by quantitative morphometric analysis. Our work demonstrates that the hierarchical‐patterned Ti_40_Zr_10_Cu_34_Pd_14_Sn_2_ BMG maintains favorable mechanical properties from as‐cast materials, such as Young's modulus and hardness. In addition to hard‐tissue implant applications, TPF‐patterned Ti‐BMGs have highly ordered features and design flexibility on the surface, which can be a prospective platform for studying cell behavior on stiff and ordered surface topographies.

## 2 Results

### 2.1 Surface Topography of TPF‐Patterned Ti‐BMGs

**Figure 1:**
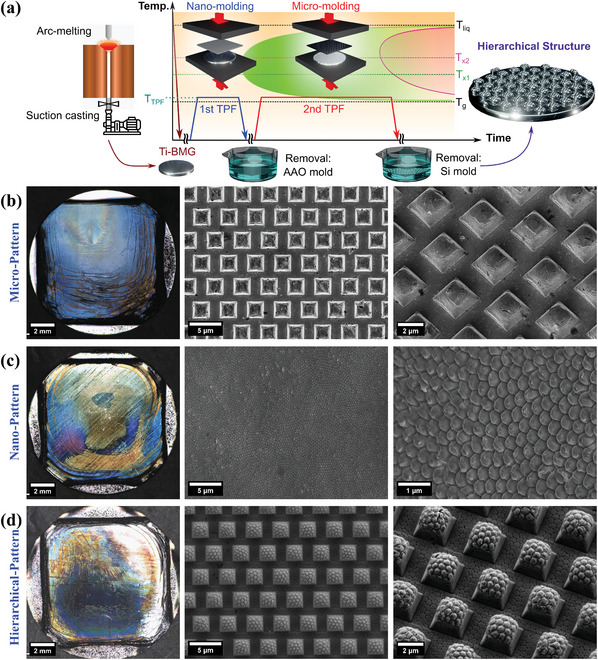
Thermoplastic forming of Ti_40_Zr_10_Cu_34_Pd_14_Sn_2_ BMG: a) Demonstration of two‐step TPF process combined with a schematic time‐temperature‐transformation (TTT) diagram. Actual samples of b) micro‐pattern, c) nano‐pattern, and d) hierarchical‐pattern with raspberry‐like appearance. The photos of the whole BMG disk are on the left, while SEM images are on the middle and right.

Ni‐free Ti‐BMG systems have been patterned into nano‐scale, and the two‐tier hierarchical structure integrating micro‐ and nano‐patters is achieved (**Figure** **1**). A demonstration of the two‐step TPF process combined with a schematic time‐temperature‐transformation (TTT) diagram is exhibited in Figure 1a. The glass transition temperature is at *T*_g_ (394 ± 2 °C), and the processing temperature is represented as *T*_TPF_. The green crystallization nose represents the first crystallization event at *T*_x1_ (446 ± 2 °C), and the pink crystallization nose represents the second crystallization at *T*_x2_ (525 ± 2 °C). The thermoformed patterns were inspected by SEM (Figure 1). The micro‐pattern (Figure 1b) has ordered square protuberances with 2.5 µm in length and around 1.5 µm in height. The nano‐pattern (Figure 1c) features 400 nm hexagonal close‐packed protrusions, similar to the corneal surface structures of insect eyes.^[^ ^49^ ^]^ The hierarchical pattern (Figure 1d) combines the topographies of micro‐pattern and nano‐pattern, giving ordered raspberry‐like hierarchical structures. The hierarchical structure is created by a two‐step TPF process in which the nano‐pattern (Figure 1c) is imprinted during the first step, followed by the generation of the micro‐pattern (Figure 1b), which is superposed on the nano‐pattern. Therefore, each “‘raspberry”’ is based on 2.5 µm square protuberances and possesses 400 nm diameter, hexagonal close‐packed protrusions on the top, leading to a total height of ≈2 µm. In the hierarchical structure, each 2.5 µm square protuberance has an average of 25 pieces of 400 nm diameter nano‐protrusions, as is illustrated in Figure S1 (Supporting Information).

### 2.2 Structural and Mechanical Properties in Terms of Thermal History

The states of BMGs depend on their thermal history.^[^ ^50^, ^51^ ^]^ The differences in states after the various TPF processing techniques were evaluated. **Table** **1** describes the terminologies, color codes, intended applications used in the following figures, and the corresponding thermal histories of the TPF conditions. “‘As Cast”’ and “‘Crystalline”’ are references in which “‘As Cast”’ represents a fully amorphous state without thermo‐processing, and “‘Crystalline”’ represents the same material annealed to a fully crystallized structure. “‘410°C_10m”’ stands for the TPF process at 410°C for 10 min. This condition is used to generate micro‐ or nano‐patterns in this work. “‘418 °C_4M +410 °C_10m”’ means the two‐step TPF process to fabricate a 2‐tier hierarchical structure in which the material is held at 418 °C for 4 min to imprint the nano‐pattern in the first step, and then at 410 °C for 10 min to superpose the micro‐pattern in the second step. “‘410 °C_10M +374 °C_6H”’ is the thermal history of a diffusion‐based nano‐molding technique inspired by Liu et al. to create nanorods with less than 100 nm diameter on crystalline metals.^[^ ^52^ ^]^ The idea is to create the nano‐protrusion at 410 °C for 10 min via the TPF process and then drop the temperature to 374 °C for 6 h while holding the same pressure all the time. “‘440 °C_4m”’ is based on the previous work from Cai et al. using the same BMG system that allows the nanocrystallization during the TPF process.^[^ ^20^ ^]^

**Table 1:** Thermal history, color code, and intended application for various TPF processing techniques. (Format for thermal history: Temperature°C_Time, M = minute; H = hour)

Thermal History	Color	Intended Application
As Cast	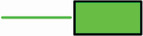	Reference (fully amorphous)
410 °C_10m	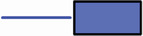	Micro‐ / Nano‐ pattern
418 °C_4m +410 °C_10M	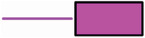	2‐tier hierarchical structure
410 °C_10m +374 °C_6H	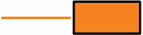	TPF + Diffusion‐based Nano‐molding
440 °C_4m	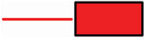	TPF with nano‐crystallization
Crystalline	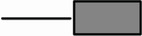	Reference (fully crystallized)

**Figure 2:**
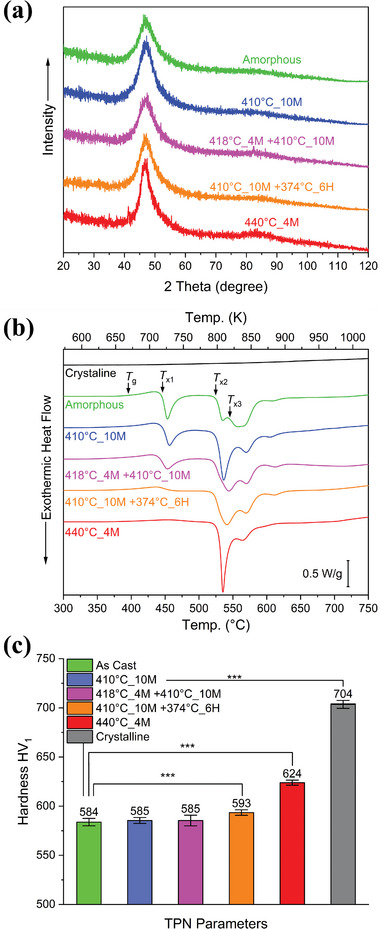
Characterization of Ti_40_Zr_10_Cu_34_Pd_14_Sn_2_ BMG disks with various thermal histories during the TPF process: a) XRD analysis, b) DSC curves from constant‐rate heating (20 °C min^−1^) measurements, and c) Microhardness.

TPF‐processed samples were characterized by XRD analysis to determine their microstructures. The XRD patterns of all TPF‐processed samples are similar to the as‐cast sample (**Figure** **2**a), with the only feature being the characteristic broad halo around 48°. None of the XRD patterns reveal obvious crystalline peaks, suggesting the amorphous structure was retained. However, it should be noted that XRD may not detect nanocrystalline phases or small‐volume fractions of crystals.^[^ ^6^, ^53^ ^]^ Nevertheless, the results of XRD analysis suggest that thermal processing in all conditions tested here does not induce significant crystallization. Figure 2b shows the results of the isochronous DSC analysis. For the references of the two extreme cases, the green line represents the amorphous state of the as‐cast sample, and the black line represents the crystalline state. Three exothermic peaks are observed in the amorphous curves, indicating the formation of crystalline phases. It has been reported that the first exothermic peak is due to the formation of α‐(Ti/Zr), Pd_3_Ti, CuTi_2_, and Pd_2_Ti crystalline phases; the second peak to the formation of the CuTi crystalline phase; the third peak has been associated with the formation of the Pd_5_Ti_3_ and CuTi_2_ crystalline phases.^[^ ^54^ ^]^ Notably, the curves for 410 °C_10M (blue) and 418 °C_4M +410 °C_10M (purple) show the occurrence of the first exothermic peak. However, the first exothermic peak in these samples is less intense than in the as‐cast sample. In the DSC curve of 440 °C_4M (red), the first exothermic peak disappeared. According to Calin et al.^[^ ^25^ ^]^ the first crystallization event involves forming a nanocrystalline α‐(Ti/Zr) phase. Hence, nanocrystallization occurred after the thermal processing of 410 °C_10M + 374 °C_6H and 440 °C_4M.

One of the main interests in BMGs for biomedical applications are their mechanical properties.^[^ ^55^ ^]^ The effects of the TPF processes on the mechanical properties have been evaluated here by microhardness and RUS. The Vickers hardness (HV1) results are shown in Figure 2c. The hardness of the as‐cast sample (584 HV) is similar to those found in 410 °C_10M and 418 °C_4M +410 °C_10M samples (585 HV). The hardness of 410 °C_10M +374 °C_6H increases slightly to 593 HV. The hardness of 440 °C_4M rises to 624 HV, 7% higher than the as‐cast, an increase consistent with typical values associated with relaxation in BMGs. The hardness of the fully crystallized sample (704 HV) is 21% higher than the as‐cast.

**Figure 3:**
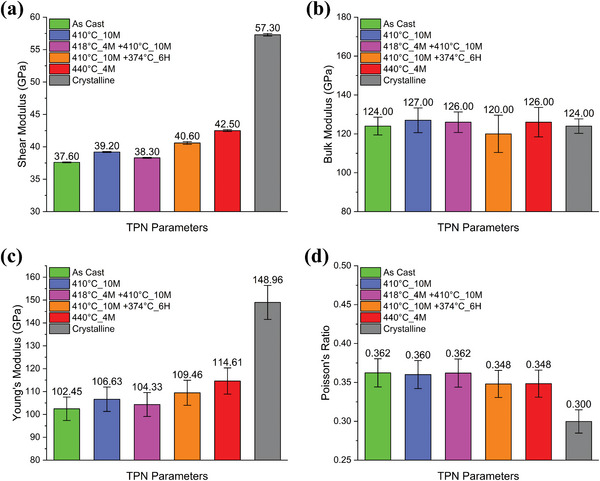
Mechanical properties determined via RUS: a) Shear modulus. b) Bulk modulus. c) Young's modulus. d) Poisson's ratio. It is worth noting that Young's modulus and Poisson's ratio were calculated from measured shear modulus and bulk modulus.

The shear and bulk modulus measured by RUS are shown in **Figure** **3**a,b. Young's modulus and Poisson's ratio were calculated from the known relationships between elastic constants in isotropic materials (Section 2.3., Figure 3c,d). The resonant modes in RUS were dominated by contributions from shear waves, and therefore, the experimental uncertainty is significantly lower for the shear modulus G (±0.2%) than for the bulk modulus K (±5%).^[^ ^56^ ^]^ The shear modulus increases gradually with harsher thermal processing conditions (longer time and higher temperature). Compared to the as‐cast sample, an apparent increase in shear modulus is observed in the processing condition of 410 °C_10M +374 °C_6H (8%), 440 °C_4M (13%), and Crystalline (52%). The bulk modulus does not change within the uncertainty following thermal processing, and thus, Young's modulus follows the same trend as the shear modulus. Compared to the as‐cast, a noticeable rise of Young's modulus is shown in 410 °C_10M +374 °C_6H (7%), 440°C_4M(12%), and Crystalline (45%). The Poisson's ratio shows an opposite trend to Young's modulus.

### 2.3 Wettability and Biocompatibility Regarding Surface Topography

**Figure 4:**
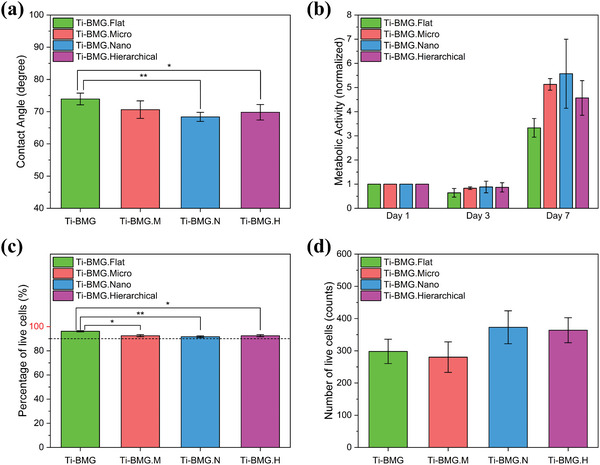
Wettability and biocompatibility of Ti_40_Zr_10_Cu_34_Pd_14_Sn_2_ BMG with flat, micro‐pattern, nano‐pattern, hierarchical‐pattern: a) Water contact angles. b) Saos‐2 cell proliferation at days 1, 3, and 7 by Alamar Blue assay. c) Percentage and d) number of live cells at day 3 by live/dead cell viability kit.

The micro‐, nano‐, and hierarchical‐patterned Ti‐BMG disks, as illustrated in Figure 1, are further investigated for their surface properties, such as wettability and biocompatibility. The original mirror‐polished Ti_40_Zr_10_Cu_34_Pd_14_Sn_2_ BMG disk was provided as a reference (Ti‐BMG.Flat). The result of contact angle measures is shown in **Figure** **4**a. All patterns result in similar wettability. The flat reference surface exhibits the largest contact angle of 74°. All patterned surfaces have contact angles slightly smaller than that. The micro‐pattern exhibits a contact angle of 71°, the hierarchical pattern a contact angle of 70°, while the nano‐patterned surface has the smallest among the four tested surfaces with 68°.

Biocompatibilities of patterned Ti_40_Zr_10_Cu_34_Pd_14_Sn_2_ BMG disks were investigated from several aspects, such as cell viability, cell proliferation, cell morphology, and cell adhesion.

**Figure 5:**
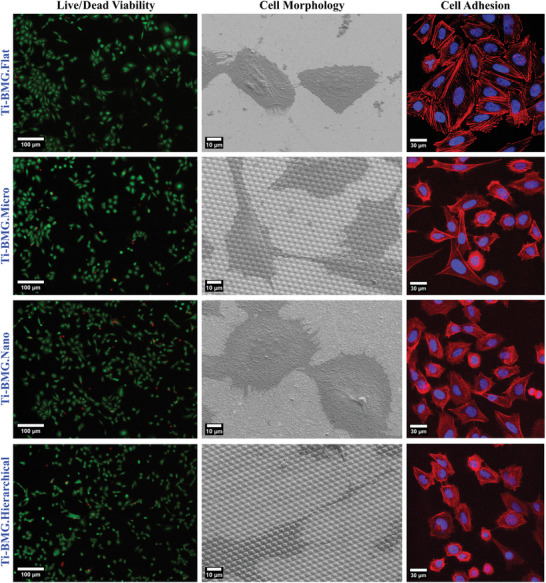
Cell viability, morphology, and adhesion of Saos‐2 cells at day 3. In the live/dead viability, live cells are stained in green, while dead cells are stained in red. In the cell adhesion, actin cytoskeleton (red) and nuclei (blue) can be observed.

Saos‐2 cell proliferation from Alamar Blue assay on four different surface topographies of Ti‐BMG was analyzed on days 1, 3, and 7. The results are displayed in Figure 4b. The metabolic activity results were normalized regarding the value of day 1 and then compared among different surface topographies on days 3 and 7. No significant differences due to the four surface topographies were observed at any time point. Cell viability was evaluated from live/dead images by analyzing Saos‐2 cells grown on the surfaces for 3 days after seeding. The quantified results, such as the percentage and number of live cells, are presented in Figure 4c,d correspondingly. All four surface topographies of Ti‐BMG have cell viability higher than 90% and the Ti‐BMG.Flat has viability even above 96%. There are no significant differences between the four surface topographies regarding the number of live cells. Representative images from the live/dead assay of each surface topography are displayed in **Figure** **5** Live/dead images, while live cells were dyed in green and dead cells in red.

Saos‐2 cell morphology and cell adhesion on the four surface topographies were investigated after 3 days of culture via SEM and CLSM, as demonstrated in Figure 5. According to SEM images, Saos‐2 cells spread well on all tested surfaces. Multiple cells are connected to each other by filopodia. Especially, filopodia longer than 20 µm are observed on the Ti‐BMG.Micro and Ti‐BMG.Hierarchical. Furthermore, these filopodia crossover the micro protuberances instead of going around them.

Regarding CLSM images from DAPI/phalloidin staining, cells on the flat surface have polygonal morphology with larger areas and are closer to each other than on patterned surfaces. Actin stress fibers were observed on all four surfaces, even though the flat surface has the most well‐defined and prolonged actin stress fibers. The cells on the three patterned surfaces have less homogenously spread cells, with some of them stretched and elongated morphology and other cells in a rounded shape. It is worth noticing that some cells on all patterned surfaces have star‐shaped or dendritic morphologies. Several dividing cells are observed on all tested surfaces.

**Figure 6:**
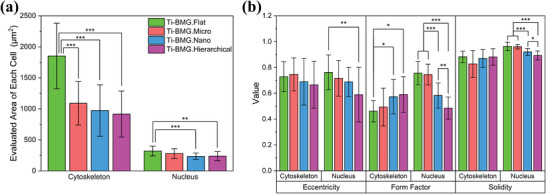
Quantitative morphometric analysis from DAPI/phalloidin staining images (Cell Adhesion in Figure 5) of Saos‐2 cells after 3 days in culture on the four surface topographies of Ti‐BMG: a) Area, b) eccentricity, form factor, and solidity of nuclei and cytoskeleton of each cell.

The CLSM images from DAPI/phalloidin staining were quantitatively analyzed. **Figure** **6**a displays the areas of the cytoskeleton and nucleus of each cell on the surfaces. Briefly, all three patterned surfaces have significantly smaller cytoskeleton areas than the flat surface, while only nano‐ and hierarchical‐patterned surfaces significantly reduce the nucleus area. In Figure 6b, three morphometric parameters: eccentricity, form factor, and solidity, are evaluated to describe cell morphologies. Each of these parameters has values ranging from 0 to 1. Tendencies were observed in the form factor. Nano‐ and hierarchical‐patterned surfaces have significantly increased values in the cytoskeleton. However, the nuclear form factor exhibits an inverse tendency, with the flat surface having the highest value and the hierarchically patterned surface having the lowest value. More interpretation about morphologic analysis is revealed in the discussion section.

## 3 Discussion

### 3.1 Influence of Thermal History on the Amorphous Structure and Mechanical Property

The correlations between the thermal histories from the TPF process, the glassy states, and mechanical properties were studied. Thermal processing of MGs at high temperatures typically induces two effects: relaxation and/or crystallization. Both relaxation and crystallization can be quantified by DSC analysis. According to crystallization enthalpies from DSC analysis, the studied TPF conditions can be divided into three categories, as listed in **Table** **2**. Category I contains the samples that still show the first exothermic peaks around 450°C in the DSC curves, for instance, As Cast, 410 °C_10M, and 418 °C_4M +410 °C_10M. Category II is for those whose first exothermic peaks disappeared, including 410 °C_10M +374 °C_6H and 440 °C_4M. Category III is Crystalline, with all three peaks and the glass transition event having vanished.

**Table 2:** Three categories according to crystallization enthalpy analysis from DSC curves in Figure 2b

Category	Thermal History	Crystallization Enthalpy 1 [J/g] ± 5%	Crystallization Enthalpy 2+3 [J/g] ± 5%
I	As Cast	8.0	28.1
I	410°C_10M	7.0	29.6
I	418 °C_4M +410 °C_10M	5.2	26.4
II	410 °C_10M +374 °C_6H	NA	28.3
II	440 °C_4M	NA	26.9
III	Crystalline	NA	NA

It is clear that if the first exothermic peak in the DSC curves is retained after the thermal processing, as for Category I, then the mechanical properties also do not change significantly. The Vickers hardness results (Figure 2c) show no significant difference between the as‐cast sample and the sample 410 °C_10M and 418 °C_4M +410 °C_10M. However, the hardness increases dramatically if the thermal processing takes longer and is harsher so that the first exothermic peaks vanish, as for Category II. Once the material is fully crystallized, as for Category III, an even higher hardness is reached.

The assessment of elastic constants is consistent with the observed hardness and state changes. Typically, the shear modulus does not change by more than a few percent in the amorphous state.^[^ ^57^ ^]^ The shear modulus in Category I is raised by less than 5%, suggesting the amorphous state is maintained. The shear modulus increases with relaxation in the glassy state have been reported in the range of 0‒7%.^[^ ^57^ ^]^ Upon crystallization, the shear modulus has been reported to increase by 15‒50%, with a typically cited value of 30%.^[^ ^58^, ^59^, ^60^ ^]^ Here, the samples assigned to Category II show shear modulus increases of 8% and 13% compared to the as‐cast sample; these values are high, suggesting a potential mixture of relaxation and some nanocrystal formation. The crystalline sample (Category III) has a massive increase in shear modulus (≈50%). The estimated Young's modulus and Poisson's ratio are influenced mainly by shear modulus since the bulk modulus weakly depends on thermal history.^[^ ^61^ ^]^

Former studies about the TPF‐based processing of BMGs claimed that crystallization must be avoided to preserve their mechanical properties.^[^ ^21^, ^51^, ^62^ ^]^ Our study suggests a crystallization tolerance for TPF processing, and slight crystallization is allowed before the Ti_40_Zr_10_Cu_34_Pd_14_Sn_2_ BMGs lose their mechanical properties. For applications where mechanical properties are considered, the system of three categories from DSC analysis can be a practical tool to find the limits of thermal processing: (i) for Category I the processed BMGs maintain the mechanical properties as in the as‐cast state, (ii) in Category II changes in mechanical properties are expected, such as higher hardness, Young's modulus, and lower Poisson's ratio, and (iii) Once the BMGs are fully crystallized as in Category III, the hardness and Young's modulus increase substantially. It is worth noting that the Crystalline sample exhibited brittle behavior during RUS sample preparation. Regarding orthopedic and dental implant applications, Ti_40_Zr_10_Cu_34_Pd_14_Sn_2_ BMGs in Category I still show relatively lower Young's modulus (104 GPa) and higher Vickers hardness (585 HV) than the Ti‐6Al‐4V alloy, where the Ti‐6Al‐4V alloy is reported Young's modulus of 110‒120 GPa and Vickers hardness 340‒350 HV.^[^ ^1^, ^14^, ^63^ ^]^

### 3.2 Interpretation of Wettability and Cell Response from Surface Topography

Wettability influences biocompatibility and can be modified through surface chemistry and topography.^[^ ^29^, ^37^, ^64^ ^]^ Generally, a surface is considered hydrophilic if the contact angle is smaller than 90° and hydrophobic if the contact angle is larger than 90°.^[^ ^9^, ^30^, ^65^ ^]^ According to the literature, hydrophilic surfaces have the advantages of upregulating bone cell attachment, spreading, differentiation, and later‐stage mineralization.^[^ ^29^, ^66^, ^67^, ^68^ ^]^ All tested surfaces of Ti_40_Zr_10_Cu_34_Pd_14_Sn_2_ BMG disks are hydrophilic since they have contact angles smaller than 75°. It is worth noting that the passive oxide layer formed on the patterned surfaces is thicker than on the flat surface due to the TPF processing temperature above 400°C. The passive oxide layer has been reported to have the composition of 64‒69% TiO_2_, 15‒20% ZrO_2_, 10‒18% Cu_2_O, and 1‒2% SnO_2_ on Ti_40_Zr_10_Cu_34_Pd_14_Sn_2_ BMG.^[^ ^6^ ^]^ Both surface topography and surface chemistry have effects on wettability.^[^ ^69^, ^70^, ^71^, ^72^ ^]^ Hence, nano‐ and hierarchical‐patterned surfaces are more hydrophilic than the flat surface due to the synergetic effect of the passive oxide layer formed during the TPF process and the patterned topographies after the TPF process. Further studies are required to determine the independent influence of surface topography and surface chemistry on wettability.

In this study, all Ti_40_Zr_10_Cu_34_Pd_14_Sn_2_ BMG disks have very high biocompatibility. Although there is no dramatic difference in cell viability and proliferation according to the Alamar Blue assay and Live/Dead Cytotoxicity, the topographical patterns have significantly changed the cell morphology and spatial distribution.

In the CLSM and SEM images (Figure 5), cells adhered to all patterned surfaces have many membrane extensions on the edge of the cell leading to star‐shaped and dendritic cell morphologies. Especially on the micro‐ and hierarchical‐patterned surfaces, these membrane extensions can expand longer than 20 µm and climb over the micro protuberances instead of going around the protuberances. These extensions are filopodia filled with bundled actin filaments and work as topographical sensors in the microenvironment.^[^ ^38^, ^69^, ^70^, ^71^ ^]^ Filopodia play important roles in driving fundamental cell functions such as cell adhesion, spreading, migration, and even division.^[^ ^38^, ^69^ ^]^ Many works^[^ ^30^, ^38^, ^72^, ^73^ ^]^ report that patterned surfaces promote the formation of longer filopodia, as observed in the CLSM and SEM images. Moreover, some cells develop filopodia parallel to each other to align with the direction of the micro‐pattern, and several nuclear signals have black spots (spots with weak fluorescence signal) ordered in the nucleus, as shown in Figure S2 (Supporting Information). The black spots observed in the nucleus were deformations of the nucleus due to the micro‐pattern. Generally, Saos‐2 cells spread well on all tested surfaces in the CLSM images (Figure 5). The quantitative morphometric analysis provides a clear view of the influence of surface topography on cell morphology (Figure 6). From the area of the cytoskeleton and nucleus (Figure 6a), a ratio of cytoskeleton area to nucleus area can be calculated, which simply indicates how well cells spread on the surface. The flat surface has the highest ratio of 5.86, while the hierarchical‐patterned surface has the lowest ratio of 3.80. The cell spreading results revealed that cells growing on the flat surface had the largest spreading area. For micro‐ and hierarchical‐patterned surfaces, the area was 1090.45 µm^2^ versus 916.97 µm^2^, respectively. These numbers indicate that each cell covered approximately 62 pieces of square protuberances for micro‐patterned surfaces and 52 pieces for hierarchical‐patterned surfaces, taking into account the protuberance size of 2.5 × 2.5 µm^2^. Several studies have shown that a limited spreading area of cells is beneficial to maintain the undifferentiated state of embryonic stem cells.^[^ ^74^, ^75^ ^]^

The morphologies of the cytoskeleton and nucleus are described using three morphometric parameters: eccentricity, form factor, and solidity. First, eccentricity measures how much the cell deviates from being circular using the bounding ellipse.^[^ ^76^, ^77^ ^]^ A perfect circle has an eccentricity of 0, and a more elongated shape has a higher eccentricity.^[^ ^76^, ^77^ ^]^ It is worth noticing that the nuclear eccentricity on the hierarchical‐patterned surface is significantly lower than on the flat surface, which implies that the nuclei are more elongated on the flat surface than on the hierarchical‐patterned surface. Rangamani et al. showed that increasing nucleus eccentricity enhances mitogen‐activated protein kinase (MAPK) 1,2 level in the COS‐7 cell nucleus for growth factor receptor pathways.^[^ ^78^ ^]^ Form factor, also known as circularity, represents how starfish‐like the shape (i.e., more protrusions and lobulations) compared with a circle.^[^ ^77^, ^79^ ^]^ A perfect circle has a form factor of 1, and the ratio decreases with more cell protrusions or deconvoluted shapes.^[^ ^77^, ^79^ ^]^ The cytoskeleton form factor of all four surfaces is smaller than 0.6, indicating that cells spread with several protrusions. Furthermore, the cytoskeleton form factor significantly increases on nano‐ and hierarchical‐patterned surfaces. However, the nuclear form factor decreases in nano‐ and hierarchical‐patterned surfaces (0.58 versus 0.49) compared with the flat surface (0.76). This decreasing trend indicates the nano‐feature ridging the nucleus, and this effect can be amplified when the micro‐feature is present, as shown in the hierarchical‐patterned surface. Lastly, solidity is the ratio of the measured area to the area of a convex hull drawn around the object.^[^ ^77^, ^80^ ^]^ This convex hull around the object can be imagined as a rubber band stretched around the perimeter so that the convex hull gaps away from the actual perimeter in undulating cells.^[^ ^77^, ^80^ ^]^ Solidity is 1 if the actual perimeter is the same as the convex hull, and solidity is smaller if there are more retracted gaps of the actual perimeter than the convex hull. Nuclear solidity has a similar trend as the nuclear form factor, with no differences between flat and micro‐patterned surfaces (0.96). Nevertheless, the nuclear solidity decreases to 0.92 for nanopatterned surfaces and 0.89 for the hierarchical‐patterned surface. Again, it is interesting that the micro‐pattern alone does not crumple the nucleus, while the nano‐pattern does. Nevertheless, when combined with the nano‐pattern, the micro‐pattern creates a more irregular nuclear shape.

Regarding cell spatial distribution, in the CLSM images, it is apparent that Saos‐2 cells are packed closer to each other on the flat surface than on three patterned surfaces. When comparing multiple live/dead images, Saos‐2 cells on the flat surface tend to form colonies in some regions and leave empty spaces in other regions. On the contrary, especially on the hierarchical‐patterned surface, Saos‐2 cells tend to disperse on the whole region with lesser cells tightly contact with each other than on the flat surface. The role of surface topography on protein absorption and subsequent cell response needs to be further elucidated. Studies showed that micro‐roughness increases protein adsorption by providing a larger specific surface area due to more active sites and features, while the aspect ratio of nano‐roughness can affect protein conformation and orientation.^[^ ^81^, ^82^, ^83^, ^84^ ^]^ A possible hypothesis is that the topography of patterned surfaces hinders cell migration. Therefore, cells develop long filopodia to sense each other instead of moving to the neighbor cells. Dallas et al. proposed that changes in cell motility may be critical in the transition from osteoblast to osteocyte, as reflected in the altered expression of many molecules involved in cytoskeletal function.^[^ ^85^ ^]^ Hence, combining cell culture on patterned surfaces with dynamic imaging approaches could be used to study the role of cell motility in bone cell function.

This work suggests potential applications of thermoplastic‐formed Ti_40_Zr_10_Cu_34_Pd_14_Sn_2_ BMG in two aspects: (1) Hard‐tissue implants and (2) a toolbox for studying cell response. Regarding implant applications, the two‐step TPF process successfully creates hierarchical structures on Ti_40_Zr_10_Cu_34_Pd_14_Sn_2_ BMGs, and most importantly, the mechanical properties, such as Young's modulus and hardness, are retained. This work confirms that the surface patterns on the Ti_40_Zr_10_Cu_34_Pd_14_Sn_2_ BMG disks do not deteriorate their biocompatibility. Furthermore, several studies demonstrate that micro‐ and nano‐scale topographies can reduce bacteria adhesion, thereby improving the antibiofilm properties of the material.^[^ ^86^, ^87^ ^]^ Therefore, further research to investigate the antibacterial properties of these patterned surfaces is crucial for hard‐tissue implant applications. The patterned BMG surfaces created by the TPF process show a high degree of order (instead of random roughness) and design flexibility, which are beneficial to study cell behavior such as spreading, adhesion, proliferation, migration, and differentiation. Currently, most patterns with high degree of order are created on polymeric substrates, and fewer studies investigated cell responses on rigid‐ and ordered‐patterned surfaces.^[^ ^29^ ^]^ Compared with random roughness, surface topography with well‐defined dimensions can easily qualify and quantify the effect of a specific cell response from a particular surface feature.^[^ ^40^ ^]^ Several reviews have discussed how pattern parameters – such as the aspect ratio, repeat space, and shape of a patterned surface – can significantly affect cell proliferation, adhesion, migration, and differentiation with different cell types.^[^ ^29^, ^36^, ^88^, ^89^, ^90^, ^91^ ^]^ In general, micro‐structured substrates can affect cell morphology and cytoskeletal structure, while nano‐structured surfaces can influence cell functions, including proliferation, differentiation, and alignment.^[^ ^90^ ^]^ The surface features of the hierarchical structures can be adjusted by implementing molds with different pore sizes and interpore distances. The prominence of a particular feature can be tuned through the applied pressure and holding time during the TPF. This design flexibility allows optimizing the particular surface feature to strengthen the desired response and combining multiple functions using a hierarchical structure.

## 4 Conclusion

Nano‐patterns and multitier hierarchical structures have been fabricated with Ni‐free Ti‐based BMGs. The versatility of the TPF technique is exhibited on Ti_40_Zr_10_Cu_34_Pd_14_Sn_2_ BMG. With 400 nm hexagonal close‐packed nano‐protrusions, the nano‐pattern mimics the microstructure of a dragonfly's eye. A two‐step TPF process takes the surface patterning to the next level by creating a hierarchical structure integrating 400 nm nano‐protrusions on 2.5 µm square protuberances. The hierarchical‐patterned Ti‐BMG preserves advantageous mechanical properties after the two‐step TPF process. Moreover, the connection between structural and mechanical properties, such as Young's modulus and hardness, is classified into three categories based on DSC analysis. The preserved low Young's modulus is essential to alleviate stress‐shielding effects for hard‐tissue implants.

Most importantly, for biomedical applications, the biocompatibilities of flat, micro‐patterned, nano‐patterned, and hierarchical‐structured Ti_40_Zr_10_Cu_34_Pd_14_Sn_2_ BMG are investigated using Saos‐2 cell line. In‐vitro assays confirm good cell viability and cell proliferation. Despite the changes in wettability being minor, surface patterns indeed change cell behaviors such as cell morphology and adhesion. Compared with the flat surface, patterned surfaces promote the formation of longer filopodia, leading to star‐shaped and dendritic cell morphologies. Regarding cell spatial distribution, Saos‐2 cells on a flat surface pack more closely to form colonies in some areas and leave empty spaces in others. However, Saos‐2 cells scatter across the entire surface on the hierarchical‐patterned surface, with fewer cells in close contact. Beyond hard‐tissue implant applications, TPF‐patterned Ti‐BMGs provide a high degree of order and design flexibility on the surface topography, which can be a prospective toolbox for exploring cell behavior on stiff and ordered surfaces.

## 5 Experimental Section

### Fabrication of Ti‐BMG Disks

Ti_40_Zr_10_Cu_34_Pd_14_Sn_2_ specimens were produced via arc‐melting and suction casting. Master ingots with nominal composition Ti_40_Zr_10_Cu_34_Pd_14_Sn_2_ (at%) were prepared from pure elements (purities above 99.9%). The ingots were melted five times to ensure homogeneity. The master ingot was cast into a disk with a 12 mm diameter and 2.5 mm thickness by copper mold suction casting. Both master ingot preparation and suction casting were carried out under a Ti‐gettered Ar atmosphere in the same arc‐melting machine (Edmund Bühler GmbH). The as‐cast Ti‐BMGs disks were cut to half the thickness using a wire‐cut electrical discharge machining (EDM MV1200S, Mitsubishi Electric Europe B.V.). The disks were ground and mirror‐polished to a thickness of ≈1 mm and roughness of less than 1 µm to eliminate potential surface crystallization and oxidation, and to ensure the surfaces were parallel for the TPF processing.

### Thermoplastic Forming of Ti‐BMG

The apparatus used for TPF in this work is inspired by the previous works by J. Schroers and collaborators at Yale University.^[^ ^92^, ^93^ ^]^ The setup is based on a compression machine (Zwick Z100, ZwickRoell GmbH & Co. KG) with ad‐hoc components, including upper and lower anvils fitted with heating cartridge, a PID controller for process control, and water‐cooling circulation to protect the loading cells from the heat. Various patterns from micro‐scale to nano‐scale, and hierarchical structures containing both, were manufactured on the Ti_40_Zr_10_Cu_34_Pd_14_Sn_2_ BMG disks. Micro‐patterns were imprinted using macro‐porous silicon templates (Si mold) with 2.5 µm pore diameter and 4.2 µm interpore distance (SmartMembranes GmbH). Nano‐patterns were imprinted from nano‐porous alumina templates (AAO mold) with 400 nm pore diameter and 480 nm interpore distance (SmartMembranes GmbH). Silicon templates were dissolved with a KOH solution (1.5 m), while alumina templates were dissolved with a phosphoric acid solution (5% w/w). Hierarchical structures were fabricated via a two‐step TPF process which created the nano‐pattern first, and then imprinted the micro‐pattern on top of the nano‐pattern as a second step (Figure S3, Supporting Information). **Table** **3** provides a summary of the processing parameters.

**Table 3:** Parameters of TPF processes to create patterns

	Step	Max. Pressure [MPa]	Load Rate [MPa min^−1^]	Load Time [Minute]	Hold Time [Minute]
Micro‐pattern	‐	900 ± 10	150	6	4
Nano‐pattern	‐	900 ± 10	900	1	9
Hierarchical structure	1	900 ± 10	900	1	9
	2	900 ± 10	150	6	4

### Characterization of Physical Properties and Wettability

The material properties of Ti‐BMG disks were characterized after the suction casting and the TPF process. Several structural analyses were conducted to confirm the amorphous nature of as‐cast states and the influence of thermal processing. The Ti‐BMG disks were characterized by X‐ray diffraction (XRD) using Bruker D2 Phaser with Co Kα radiation. To inspect the formation of nanocrystals, samples of ≈20 mg mass were characterized by differential scanning calorimetry (DSC) using a NETZSCH DSC 404 F1 instrument under an argon atmosphere at a heating rate of 20 K min^−1^ (constant‐rate heating mode) using alumina crucibles. Overview images of the sample disk were recorded with confocal laser scanning microscopy (CLSM‐Olympus LEXT OLS4100). The topographies of patterned surfaces were imaged with scanning electron microscopy (SEM‐ Zeiss LEO type 1525) using an in‐lens detector. Microhardness was assessed with standard microindentation with a Vickers indenter, and the elastic constants with resonant ultrasound spectroscopy (RUS). Following ISO 6507 standard, Vickers hardness tests were performed using a 9.8 N load (HV1) on a DuraScan 70 G5 universal laboratory hardness tester (EMCO‐TEST Prüfmaschinen GmbH, Austria). RUS was carried out at room temperature (RT) on cuboid samples in the as‐cast state and after the various TPF conditions. The methodologies employed to determine the values of the elastic moduli were identical to those of McKnight et al.^[^ ^88^ ^]^ A lapping fixture was used to ensure the parallelism and perpendicularity of the faces. The root mean square (RMS) error of the fitting to the resonance spectra were <0.85%. The given Young's moduli and Poisson's ratios were computed from the measured bulk modulus and shear modulus using conversion Formulas (1) and (2), respectively:

(1) $$E\;=\frac{9\;K\;G}{3K+G}$$

(2) $$\nu \;=\frac{3K-2G}{2\left(3K+G\right)}$$

where *K* is the bulk modulus, *G* is the shear modulus, *E* is Young's modulus, and *ν* is Poisson's ratio. Static water contact angles were recorded and analyzed via the sessile drop method with a contact angle goniometer (Kruess DSA100). The measurements were performed and averaged for at least five locations, with water droplets of 2 µL volume.

### Cell Culture and Cell Seeding

In vitro experiments were used to examine the four different surface topographies of Ti_40_Zr_10_Cu_34_Pd_14_Sn_2_ BMG disks: 1) Flat (mirror‐polished), 2) Micro‐pattern, 3) Nano‐pattern, 4) Hierarchical‐pattern. Osteoblast‐like Saos‐2 cell line (ATCC HTB‐85), derived from primary human osteosarcoma, were cultured in the fresh medium composed by Dulbecco's modified Eagle medium (DMEM) with 10% fetal bovine serum (Gibco) and 2% Penicillin‐Streptomycin (P/S; Biowest, Riverside, MO, USA), under standard conditions (37 °C and 5% CO_2_). All Ti‐BMG disks were sterilized with absolute ethanol for 30 min and air dried. The sterilized disks were placed into a 24‐well plate individually. Then, 20 000 Saos‐2 cells were seeded on top of the disks in each well and cultured under standard conditions.

### Cell Viability and Cell Proliferation Assays

Cell viability on the Ti‐BMG disks was assessed using the Live/Dead Viability/Cytotoxicity kit for mammalian cells (Invitrogen, Waltham, MA, USA). Saos‐2 cells were seeded on the Ti‐BMG disks as described in Section 2.4.1. After 3 days of incubation, the assay was performed following the manufacturer's protocol. Live/dead images of eight random regions within the whole Ti‐BMG disk were captured by an Olympus IX71 inverted microscope with epifluorescence. Live/dead images were analyzed using CellProfiler (4.2.5) software to systematically count the number of live and dead cells. Saos‐2 cell proliferation was evaluated by performing an Alamar Blue cell viability test (Thermo Fisher Scientific, USA) at incubation days 1, 3, and 7. After 24 h of cell seeding, Ti‐BMG disks with adhered cells were transferred to a new well to discard the cells growing on the bottom of the well. Fresh medium with 10% Alamar Blue was added into each well, and cells were incubated under standard conditions in the dark for another 4 h. The supernatant was then collected, and fresh medium was added to the well plate to keep the culture going. 200 µL of the supernatant was transferred to a black‐bottom Greiner CELLSTAR 96‐well plate (Sigma–Aldrich, Saint Louis, MO, USA), and the fluorescence was determined at 590 nm wavelength after excitation at 560 nm on a Spark multimode microplate reader (Tecan, Männedorf, Switzerland). The assay was repeated after 3 and 7 days. Fluorescence values on days 3 and 7 were normalized to values obtained from day 1. Experiments were performed in triplicate, and each has two technical replicas.

### Cell Morphology and Cell Adhesion Analysis

The same samples from the cell viability assay were prepared for SEM imaging (Zeiss Merlin) with SE2 detector to study the influence of pattern designs on cell morphology. Ti‐BMG disks with adhered cells were rinsed twice in phosphate‐buffered saline (PBS), fixed in 4% paraformaldehyde (Sigma) in PBS for 45 min at RT, and washed twice in PBS. Cell dehydration was performed by increasing ethanol concentrations (50, 70, 90, and twice 100%) for 5 min each. Lastly, samples were dried using hexamethyldisilazane (Electron Microscope Science) for 15 min and were ready for SEM imaging. Cell adhesion was inspected through the actin filaments distribution. Saos‐2 cells were seeded on the Ti‐BMG disks as described in Section 2.4.1. After 3 days of incubation, the actin cytoskeleton was labeled with phalloidin (Invitrogen), while nuclei were stained with DAPI (Invitrogen). The staining was performed following the manufacturer's protocol. Fluorescence images were taken using a confocal laser scanning microscope (CLSM, Confocal Leica SP5, Leica Microsystems GmbH). One disk was analyzed for each surface topography. Quantitative morphometric analysis from the confocal images was performed using CellProfiler (4.2.5) software to evaluate the area, eccentricity, form factor, and solidity of cytoskeleton and nucleus.

### Statistical Analysis

Vickers hardness and contact angle are presented as a mean ± standard deviation (SD). Shear and bulk modulus from resonant ultrasound spectroscopy are presented as a mean ± uncertainty. Results from the Alamar Blue assay and live/dead cell viability kit are presented as a mean ± standard error (SE). Quantitative morphometric analysis from DAPI/phalloidin staining images is presented as a mean ± standard deviation (SD). All statistical analyses were evaluated by one‐way analysis of variance (ANOVA) and Tukey post hoc comparison using the software OriginPro 2019b (9.6.5). Differences were considered statistically significant for p‐values <0.05. (denoted by: ^*^ *p* <0.05; ^**^ *p* <0.01; ^***^ *p* <0.001)

## Conflict of Interest

The authors declare no conflict of interest.

## Supporting information

Supporting Information

## Data Availability

The data that support the findings of this study are available from the corresponding author upon reasonable request.
