# Supplementary material for: Hierarchical Surface Pattern on Ni‐Free Ti‐Based Bulk Metallic Glass to Control Cell Interactions
Source: Small. 2023 Dec 18;20(22):2310364. doi: 10.1002/smll.202310364 (PMC11475312; doi:10.1002/smll.202310364)
Supplement: Supplementary file 1 — Supporting Information [file SMLL-20-2310364-s001.pdf]

NANO · MICRO
small

Supporting Information

for *Small*, DOI 10.1002/smll.202310364

Hierarchical Surface Pattern on Ni-Free Ti-Based Bulk Metallic Glass to Control Cell Interactions

Fei-Fan Cai, Andreu Blanquer, Miguel B. Costa, Lukas Schweiger, Baran Sarac, A. Lindsay Greer, Jan Schroers, Christian Teichert, Carme Nogués, Florian Spieckermann* and Jürgen Eckert*

Supporting Information

Multitier Architectured Ni-free Ti-based Bulk Metallic Glass with Controllable Cell Interactions

Fei-Fan Cai, Andreu Blanquer, Miguel B. Costa, Lukas Schweiger, Baran Sarac, Jan Schroers, Christian Teichert, Carme Nogués, Florian Spieckermann, Jürgen Eckert*

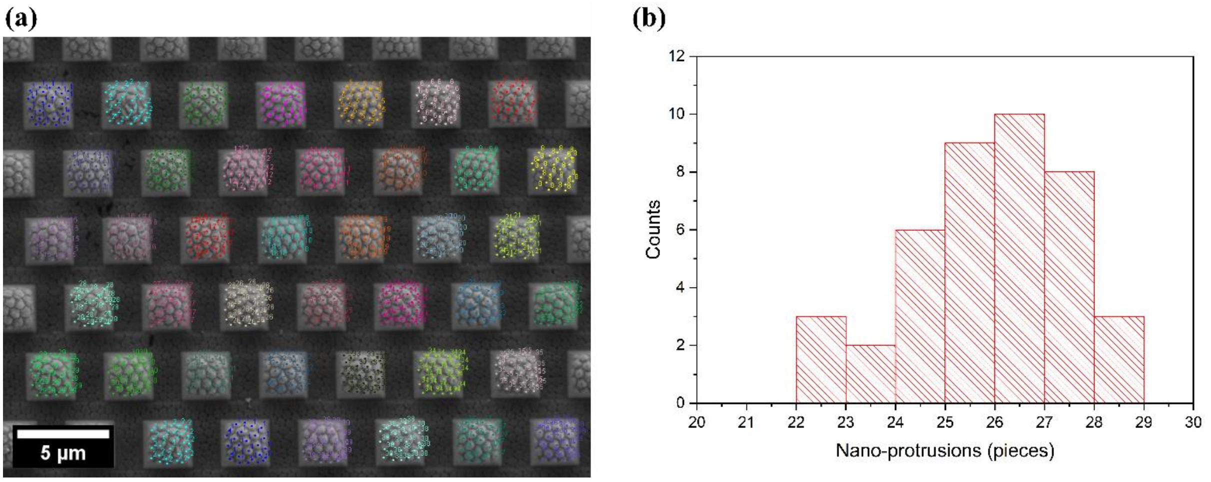

Figure S1: Nano-protrusions on square micro-protuberances for the hierarchical structure: (a) Counts via ImageJ (Fiji 1.54d). (b) Histogram of counting results.

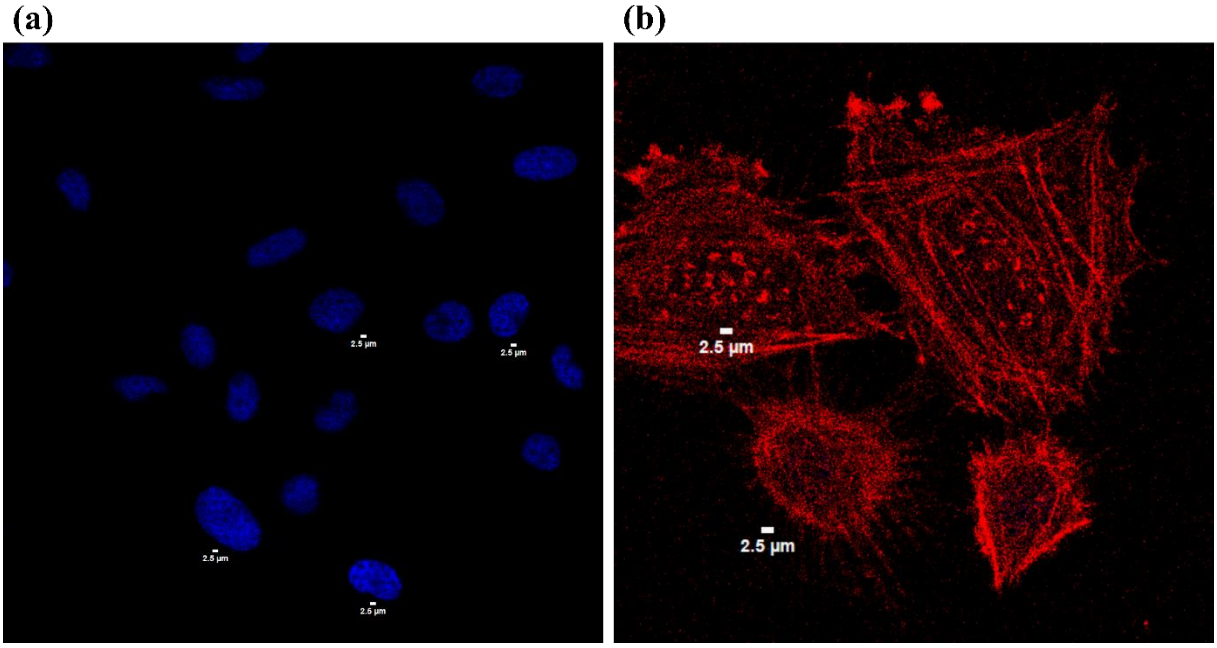

Figure S2: CLSM images of Saos-2 cells on the micro-patterned sample: (a) Spots with weak fluorescence signal (black spots) were deformations of the nucleus due to the micro-pattern.

(b) Some cells develop filopodia parallel to each other to align with the direction of the micro-pattern.

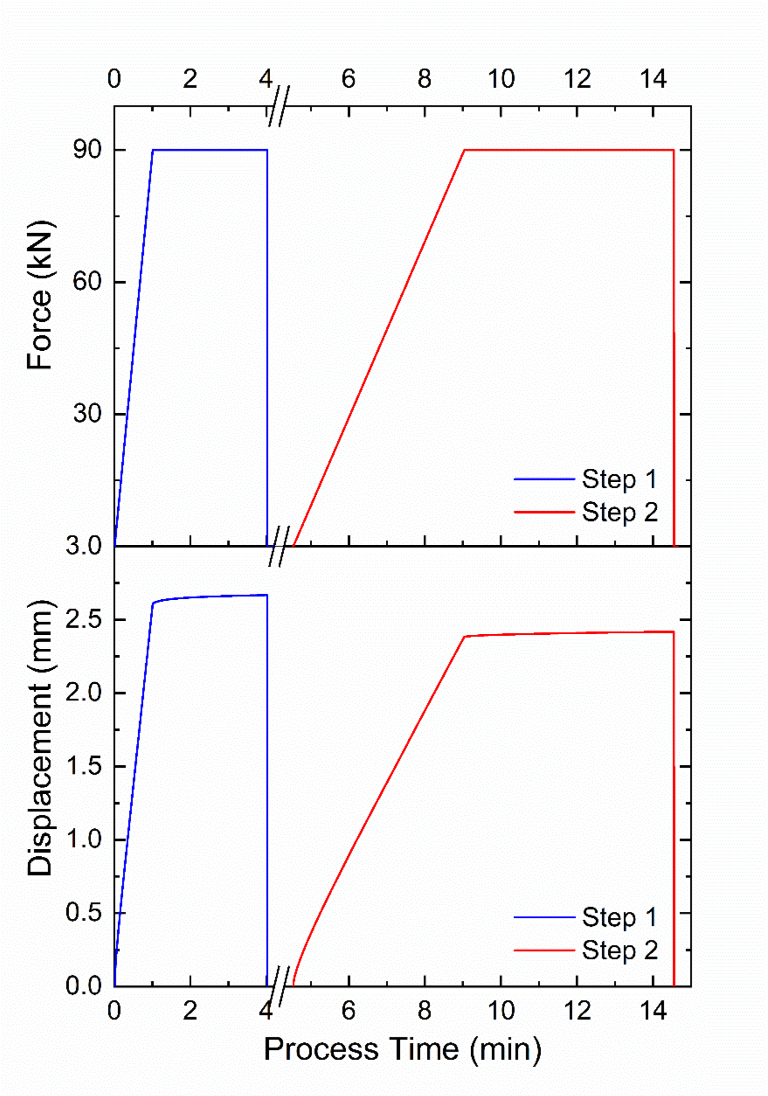

Figure S3: Record of the process parameters (applied force and displacement) for the thermoplastic forming (TPF) process of the hierarchical structures. The displacement includes the thermal expansion of the compression test machine.
